# Effects of selenium supplementation on concurrent chemoradiotherapy in patients with cervical cancer: A randomized, double-blind, placebo-parallel controlled phase II clinical trial

**DOI:** 10.3389/fnut.2023.1094081

**Published:** 2023-02-02

**Authors:** Mei Yang, Bo Pei, Qiancheng Hu, Xiaoying Li, Xiping Fang, Xue Huang, Zunjing Yang, Jiaquan Chen, Du He, Guogen Sun, Peng Lv, Li Wang, Zixiong Zhang, Lin Lai, Chuying Huang

**Affiliations:** ^1^Department of Oncology, The Central Hospital of Enshi Tujia and Miao Autonomous Prefecture, Enshi Clinical College of Wuhan University, Enshi, China; ^2^Department of Oncology, Yunfu People's Hospital, Yunfu, China; ^3^Department of Radiation and Medical Oncology, Zhongnan Hospital of Wuhan University, Wuhan, China; ^4^Department of Abdominal Oncology, Cancer Center, West China Hospital, Sichuan University, Chengdu, China; ^5^Hubei Selenium and Human Health Institute, The Central Hospital of Enshi Tujia and Miao Autonomous Prefecture, Enshi, China; ^6^Cancer Center, Union Hospital, Tongji Medical College, Huazhong University of Science and Technology, Wuhan, China

**Keywords:** cervical cancer, chemoradiotherapy, clinical trial, hematologic toxicity, selenium

## Abstract

**Objective:**

Selenium (Se) is an essential trace element and may affect cervical cancer occurrence and progression. The association between selenium supplementation and acute toxic reactions and clinical outcomes in patients with locally advanced cervical cancer treated with concurrent chemoradiotherapy remains unclear. The aim of this study was to determine the safety profile of add-on Se yeast and assess the potential of Se to ameliorate the hematologic toxicity of concurrent chemoradiotherapy in patients with cervical cancer.

**Methods:**

Patients with Federation International of Gynecology and Obstetrics (FIGO) stage IIB cervical cancer who met all inclusion criteria were randomly assigned to either the experimental group or the control group. The experimental group received Se yeast tablets (100 μg Se, twice daily), while the control group received placebos (twice daily) for 5 weeks in total. All patients in both groups received standard treatment, including pelvic external irradiation, concurrent five cycles of chemotherapy, and brachytherapy. Measures included the incidence of myelosuppression, impairment of liver and kidney function, objective response rate (ORR), and blood Se concentrations before, during and after the treatment of the two groups.

**Results:**

A total of 104 eligible patients were enrolled in the experimental group (*n* = 50) or the control group (*n* = 54). The ORR in the experimental group and control group were 96 and 94%, respectively (*p* = 0.47). The baseline levels of blood Se before treatment in the experimental and control groups were similar (58.34 ± 17.63 μg/L and 60.21 ± 18.42 μg/L, *p* = 0.60), but the concentrations became significantly different after course completion between the two groups (76.16 ± 24.47 μg/L and 57.48 ± 14.92 μg/L, respectively, *p* < 0.01). Se dramatically decreased the incidence of grade 3 myelosuppression (48% vs. 63%, *p* = 0.034) compared to the control group. In the subgroup of patients with moderately well-differentiated cervical cancer, the incidence of thrombocytopenia induced by concurrent chemoradiotherapy was lower in the experimental group than in the control group (53.8% vs. 78.9%, *p* < 0.01). However, no difference was observed in liver and kidney injuries between the two groups.

**Conclusion:**

Supplementation with Se effectively increased blood Se levels in Se-inadequate cervical cancer patients. As an add-on to standard treatment, Se-yeast significantly decreased the hematologic toxicity of concurrent chemoradiotherapy.

## 1. Introduction

Cervical cancer is one of the most common gynecological malignant tumors, with 604,000 new cases and 342,000 deaths worldwide reported in 2020 (1). The standard treatment mode of early-stage (FIGO stage IA-IB1) patients is radical hysterectomy, lymph node dissection, and/or radiotherapy, and locally advanced stage (FIGO stage IB2-IVA) patients mainly receive platinum-based concurrent chemoradiotherapy (2). The overall incidence of genitourinary complications is reported to be between 17 and 40% in patients with concurrent chemoradiotherapy, while acute toxicity of grades 3–4 occurs in 17.1% of patients with concurrent chemoradiotherapy (3). Acute toxic reactions caused by concurrent chemoradiotherapy often require dose modifications or radiotherapy treatment suspension, which could potentially impair the curative effects of concurrent chemoradiotherapy and prolong the length of stay in the hospital. Previous studies demonstrated that intensity modulation radiotherapy significantly reduced the level of gastrointestinal and hematological toxicity in patients with cervical cancer in comparison to conventional radiotherapy (4, 5). It is imperative to search for an effective strategy to decrease the incidence of acute side effects during concurrent chemoradiotherapy in patients with cervical cancer.

Selenium is an essential trace element and has extremely important biological functions in human health. Previous studies have demonstrated that selenium compounds can enhance the effect of radiotherapy and chemotherapy (6–8). Several studies have revealed that selenium supplementation may decrease the toxicity of chemotherapy and radiotherapy (9–11). However, these studies have been constrained by the limited number of patients included and the lack of randomized controlled clinical trials, and the association between selenium supplementation and acute toxic reactions and clinical outcomes in patients with locally advanced cervical cancer treated with concurrent chemoradiotherapy remains unclear. Therefore, we conducted a randomized, placebo-controlled trial to assess whether adding selenium yeast to platinum-based concurrent chemoradiotherapy would decrease acute side effects during treatment and improve efficacy compared with placebo for patients with stage IIB cervical cancer.

## 2. Patients and methods

We conducted a randomized, double-blind, and placebo-parallel controlled clinical trial to evaluate the effects and safety of Se supplementation on concurrent chemoradiotherapy in patients with stage IIB cervical cancer. This study was approved by the Ethics Committee of the Central Hospital of Enshi Tujia and Miao Autonomous Prefecture, and all patients signed informed consent forms to participate in the study. The trial was registered at https://www.chictr.org.cn/listbycreater.aspx (ChiCTR2100043379).

### 2.1. Patient eligibility

The inclusion criteria were as follows: (1) aged from 18 to 70 years old; (2) histologically or cytologically confirmed cervical cancer and diagnosed with stage IIB cervical cancer according to the 2014 International Association of Obstetrics and Gynecology (FIGO) recommendations; (3) treatment-naïve and eligible to receive first-line concurrent chemoradiotherapy; (4) performance status (PS) score 0–1 points; (5) basically normal functions of major organs (hemogram, heart, liver and kidney), white blood count ≥3.5*10^9^/L with neutrophils ≥1.5*10^9^/L, platelet count ≥100*10^9^/L, and hemoglobin ≥90 g/L. Total bilirubin ≤1.5 times upper limit of normal (ULN) range; alkaline phosphatase (ALP) ≤2.5 times ULN, Transaminases AST and ALT ≤2.5 times ULN, serum creatinine ≤1.2 times ULN; and (6) Patients must read and understand Chinese language, adhere to the study protocol, and must provide written informed consent. The exclusion criteria were as follows: (1) complications with other serious medical diseases; (2) allergy or intolerance to cisplatin or selenium yeast; and (3) pregnancy or lactation.

### 2.2. Treatment

This double-blind trial was conducted in our hospital. Patients were randomly assigned in a 1:1 ratio to receive selenium yeast tablets (100 μg Se) or placebo twice a day. Randomization was performed using a computer-generated table of random numbers. A table of random numbers was placed in sequentially sealed envelopes. Both investigators and patients were blinded to the treatment allocation. The placebo was prepared by the pharmacy and supplied to the department of cancer labeled with the predetermined coding scheme.

All patients received pelvic external irradiation with six MV X-ray at prescribed doses of PGTV 60Gy/25F and PCTV 50Gy/25F, five times a week, and five cycles of concurrent chemotherapy: intravenous infusion of cisplatin with 30 mg/m^2^, once a week. At the later stage of radiotherapy, brachytherapy was performed twice a week using the radioactive source Iridium-192. The dose at point A was 6 or 7Gy, and the total dose reached 28Gy/4F or 30Gy/5F. Treatment was continued until unacceptable toxic effects, such as grade 3 radiation proctitis and radiation cystitis according to the Radiation Therapy Oncology Group (RTOG) classification or grade 3–4 hematological toxicities during concurrent chemoradiotherapy according to the National Cancer Institute Common Toxicity Criteria (version 3.0). Investigators could interrupt or discontinue individual trial agents to manage treatment-related toxic effects.

### 2.3. Outcomes

The primary outcome of this study was concurrent chemoradiotherapy-related hematologic toxicity. The secondary outcomes were the objective response rate and safety profile, including liver and renal toxicity.

### 2.4. Evaluation

Imaging studies (magnetic resonance imaging) were performed before and after the entire treatment to evaluate the therapeutic effects according to Response Evaluation Criteria in Solid Tumor guidelines (RECIST1.1) (12). Therapeutic effects were evaluated by complete response (*CR*), partial response (*PR*), stable disease rates (*SD*) and progressive disease (*PD*). The objective response rate (ORR) was calculated as *ORR* = (*CR* + *PR*)/total cases × 100%.

Routine blood tests, routine urine tests, and serum biochemistry tests were taken once a week during the treatment to evaluate the adverse effects according to the National Cancer Institute Common Toxicity Criteria (version 3.0). The main observational items were red blood cell count, white blood cell count, neutrophil count, platelet count, bilirubin, alanine aminotransferase and alkaline phosphatase, urea nitrogen, creatinine, and proteinuria. Whole blood selenium levels were measured before, during and at the end of treatment.

### 2.5. Measurement of whole blood selenium

Blood samples from the patients with cervical cancer were drawn between 7 am and 8 am after an overnight fast. A 2–3 ml blood sample was drawn from each patient, and the blood sample was immediately frozen at 4°C until analysis. In this study, the selenium level we measured was the whole blood selenium concentration, and we used the microwave digestion method.

### 2.6. Statistical analysis

All data were analyzed using SPSS Statistics (IBM SPSS Statistics for Windows, Version 22.0). Continuous variables were described as the mean and standard deviation for normally distributed variables. Categorical variables were described as numbers and percentages. Two-sample independent-groups *t*-tests were carried out to compare differences in means between two groups, and Pearson’s Chi-square test (*χ*^2^) or Fisher’s exact test was used to compare differences between categorical variables. A two-sided *p* value <0.05 was considered statistically significant.

## 3. Results

### 3.1. Patients

**Figure 1:**
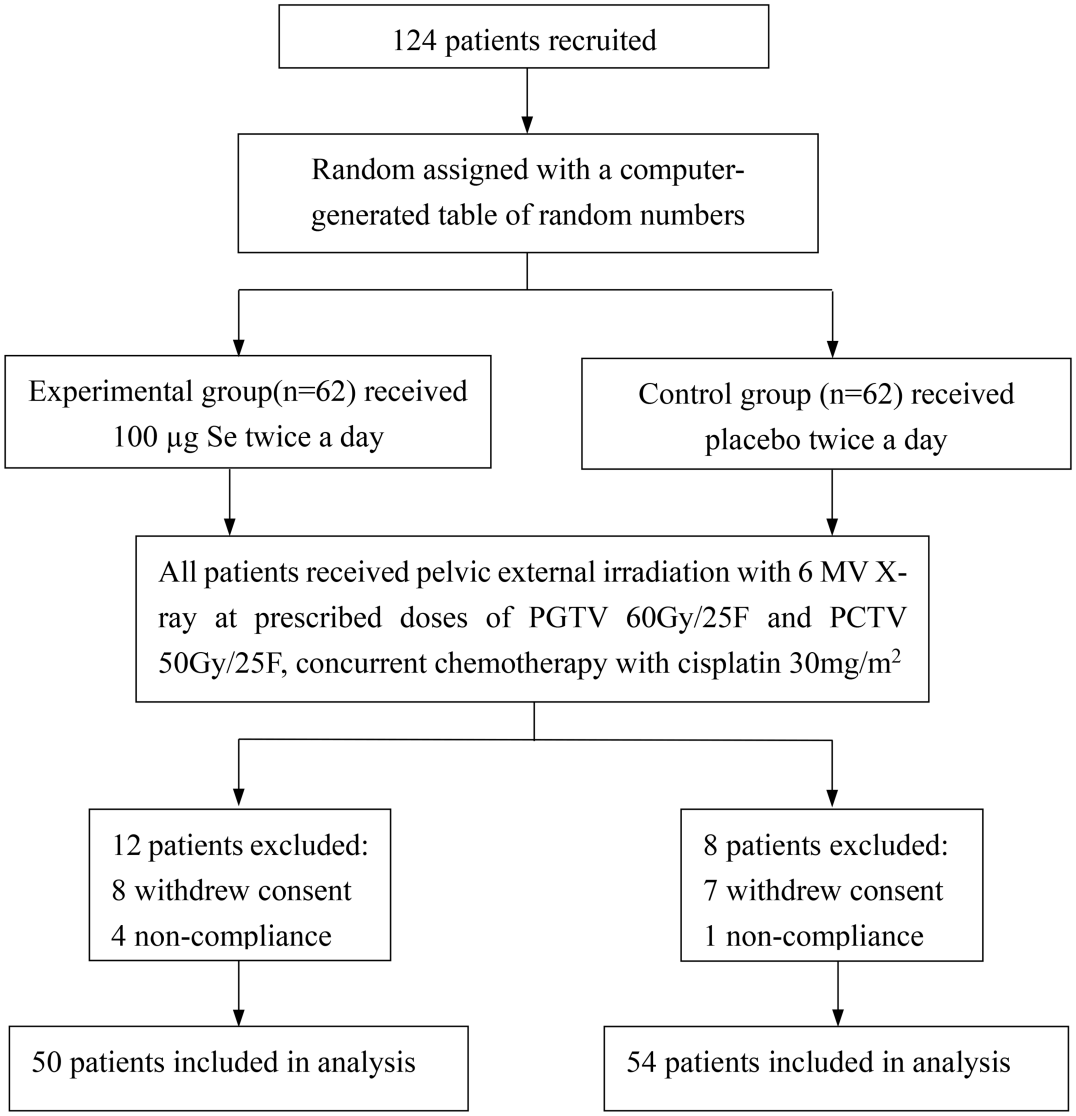
Trial profile. PGTV, planning gross tumor volume; PCTV, planning clinical target volume; CR, complete response; PR, partial response; SD, stable disease; PD, progressive disease.

From July 2018 to May 2021, a total of 124 patients were enrolled and randomly assigned to the experimental group (*n* = 62) and control group (*n* = 62), but 20 patients were excluded due to withdrawal of consent and noncompliance. Finally, 104 patients were included in the final analysis (Figure 1). There were no significant differences between the two groups in characteristics (Table 1) at baseline.

**Table 1:** Baseline demographic and clinical characteristics between the two groups.

Characteristic	Experimental group	Control group	*p*-Value
No. of patients enrolled	50	54	
Median age (±SD)	58 ± 9.26	56.5 ± 9.54	0.72
Time of pregnancy	3.24 ± 1.51	3.41 ± 1.61	0.59
Number of births	2.44 ± 0.99	2.56 ± 1.22	0.60
Mean hospital stay	55.58 ± 7.61	56.00 ± 6.20	0.76
Nation			0.17
Han	22	31	
Ethnic minorities	28	23	
Education background			0.77
Illiteracy	12	10	
Primary school	29	30	
Junior high school	8	13	
Senior high school	1	1	
Pathological types			1
Squamous cell carcinoma	48	52	
Others	2	2	
Degree of differentiation			0.31
Poor	37	35	
Moderately well	13	19	

### 3.2. Efficacy outcomes

During treatment, patients have different degrees of toxicity effects related to chemoradiotherapy. Se dramatically decreased the incidence of grade 3 myelosuppression compared to the control group (48% vs. 63%, *p* = 0.034; Table 2). The incidence of grade 1–2 myelosuppression in the experimental group was 26 (92%) compared to 25 (92.59%) in the control group (*p* = 0.695). We found no significant difference between the groups for any type of adverse events, including leukopenia, neutropenia, anemia, diarrhea, thrombocytopenia, and liver damage. There was no damage to the kidney noticed in the study (Table 2). We subsequently conducted subgroup analysis of efficacy indicators according to the degree of tumor differentiation. We found that the incidence of platelet toxicity related to chemotherapy in the experimental group was lower than that in the control group (*p* < 0.01; Table 3). The secondary efficacy outcomes of ORR between the experimental group and control group were 96 and 94%, respectively (*p* = 0.47; Table 4).

**Table 2:** Adverse events with concurrent chemoradiotherapy.

Characteristic	Experimental group (*n* = 50)	Control group (*n* = 54)	*p*
Myelosuppression			
Grade 1–2	26	25	0.695
Grade 3	24	37	0.034
Grade 4	4	3	0.708
Leukopenia			0.10
Grade 0	7	1	
Grade 1	5	5	
Grade 2	20	21	
Grade 3	18	27	
Grade 4	0	0	
Neutropenia			0.83
Grade 0	9	10	
Grade 1	14	17	
Grade 2	15	18	
Grade 3	12	9	
Grade 4	0	0	
Anemia			0.32
Grade 0	12	6	
Grade 1	16	16	
Grade 2	17	22	
Grade 3	3	8	
Grade 4	2	2	
Thrombocytopenia			0.53
Grade 0	25	26	
Grade 1	11	19	
Grade 2	9	6	
Grade 3	3	2	
Grade 4	2	1	
Liver damage			0.15
Grade 0	26	37	
Grade 1	16	14	
Grade 2	7	2	
Grade 3	1	1	
Grade 4	0	0	
Bilirubin			0.83
Grade 0	47	51	
Grade 1	3	2	
Grade 2	0	0	
Grade 3	0	0	
Grade 4	0	1	
Increased alanine aminotransferase			0.26
Grade 0	39	47	
Grade 1	4	4	
Grade 2	6	2	
Grade 3	0	1	
Grade 4	1	0	
Increased alkaline phosphatase			0.26
Grade 0	29	37	
Grade 1	17	15	
Grade 2	4	1	
Grade 3	0	0	
Grade 4	0	1	
Creatinine increased			
Grade 0–4	0	0	
Urea nitrogen increased			
Grade 0–4	0	0	
Proteinuria			
Grade 0–4	0	0	
Diarrhea	5	4	0.735

**Table 3:** The incidence of thrombocytopenia in patients with moderately well-differentiated cervical cancer between the two groups.

Characteristic	Experimental group (*n* = 13)	Control group (*n* = 19)	*p*
Thrombocytopenia			<0.01
Grade 0	6	4	
Grade 1	1	14	
Grade 2	4	0	
Grade 3	1	1	
Grade 4	1	0	

**Table 4:** Response rate to first-line concurrent chemoradiotherapy treatment.

Characteristic	Experimental group (*n* = 50)	Control group (*n* = 54)	*p*
Short-term effects			
CR	10	6	
PR	38	45	
SD	2	2	
PD	0	1	
ORR	96%	94%	0.47

CR, complete response; PR, partial response; SD, stable disease; PD, progressive disease; ORR, objective response rates.

### 3.3. Whole blood selenium level

There was no significant difference between the baseline blood selenium level and the blood selenium level in the third week of treatment between the two groups (*p* > 0.05). However, after treatment, the blood selenium concentration of the experimental group was significantly higher than that of the control group (*p* < 0.01; Table 5).

**Table 5:** Selenium levels (μg/L) between the two groups.

Characteristic	Experimental group (*n* = 50)	Control group (*n* = 54)	*p*
Baseline Se level	58.34 ± 17.63	60.21 ± 18.41	0.6
During of RT	63.98 ± 17.68	58.88 ± 17.29	0.14
End of RT	76.16 ± 24.47	57.48 ± 14.92	<0.01

RT, radiation therapy.

## 4. Discussion

Selenium is an essential trace element that is fundamentally important to human health. Epidemiological studies demonstrated that salt fortified with selenium as sodium selenite decreased the incidence of hepatocellular cancer by 35% compared to the control group (13). The Nutritional Prevention of Cancer Trial was the first double-blind, placebo-controlled intervention trial to assess whether selenium supplementation could reduce the risk of cancer. Selenium supplementation showed effects of 50% lower total cancer mortality (*p* = 0.002) and 37% lower total cancer incidence (*p* = 0.001) (14). Selenium deficiency also increases the risk of cervical cancer (15). On the other hand, selenium can reduce the harmful toxicities of radiotherapy and chemotherapy without compromising efficacy (8). In this study, we found that selenium supplementation decreased the incidence of grade 3 myelosuppression (*p* = 0.034) but had no effect on the incidence of grade 0–1 myelosuppression, with 26 (52%) in the experimental group and 25 (46.3%) in the control group (*p* = 0.695). Selenium supplementation did not decrease the incidence of thrombocytopenia in patients with cervical cancer between the two groups. However, in a subgroup of patients with highly differentiated cervical cancer, a significant difference in platelet toxicity was observed, with 7 of 13 (53.8%) in the experimental group and 15/19 (78.9%) in the control group (*p* < 0.01). An earlier *in vitro* study showed that supplementation with Se yeast alone or in combination with chemotherapeutic drugs could induce apoptosis in several tumor cell lines (16). In our study, the ORR between the experimental group and the control group was not significantly different (96% vs. 94%, *p* = 0.47), indicating that selenium supplementation has a negligible impact on the efficiency of chemoradiotherapy in cervical cancer. To the best of our knowledge, this is the first clinical trial exploring the role of the addition of selenium in the treatment of patients with cervical cancer. Although several studies have confirmed that selenium supplementation enhances the efficacy of radiotherapy and chemotherapy (17), another phase II clinical trial revealed the opposite result in patients with stage III and stage IV head and neck squamous cell carcinoma treated with selenomethionine combined with concurrent chemoradiotherapy and simple concurrent chemoradiotherapy. It was found that there was no significant difference in CR, overall survival, and progression-free survival between the two groups (17). Although the short-term efficacy of selenium yeast combined with concurrent chemoradiotherapy for cervical cancer has not improved, our data showed that the incidence of myelosuppression in the experimental group was lower than that in the control group. Similar effects have been observed by Katya’s team, who noticed that there was no increase in the toxicity of platelets, white blood cell count, neutrophils, hematocrit, and hemoglobin during the selenium supplementation period in leukemia/lymphoma patients (18). Moreover, another study has shown that the addition of 500 μg/day sodium selenite during radiotherapy can reduce the number and severity of diarrhea caused by radiotherapy (10). Recently, a Japanese study revealed that serum selenium predicts achievement of full-dose cisplatin in concurrent chemoradiotherapy for locally advanced head and neck squamous cell carcinoma. Selenium deficiency before treatment was independently associated with poor compliance with cisplatin (19). Collectively, these studies support our findings that selenium supplementation ameliorates the toxic effects of chemoradiotherapy without extra gastrointestinal side effects, providing a new strategy to improve the tolerance and compliance of patients to chemoradiotherapy.

Current studies indicate that radiation therapy and cisplatin induce lipid peroxidation and ferroptosis in cancer cell lines, human cancer samples, and the renal proximal tubules of mice (20, 21). Polyunsaturated fatty acids participate in the formation of essential lipid products that play a crucial role in activating or inhibiting platelet function (22). The platelet membrane structure is very complex with a large number of lipids, making it very sensitive to radiation-induced lipid peroxidation. Recently, a study indicated that although the exposure dose was below the limit, medical workers exposed to low-dose ionizing radiation for a short period of time might have increased first and then decreased platelets (23). Hemoglobin, heme, and hemin produced by old red blood cells trigger ferroptosis in platelets (24). GPX4 is a selenoprotein that utilizes reduced glutathione to convert lipid hydroperoxides to lipid alcohols, thereby alleviating lipid peroxidation and inhibiting ferroptosis. Selenium supplementation can activate GPX4 activity and alleviate ferroptosis inducer RSL3 and erastin-induced lipid oxidation in the retina and heart samples of mice (unpublished data). Hence, we hypothesize that selenium supplementation ameliorates the toxic effects of chemoradiotherapy on platelets by activating GPX4 and mitigates lipid peroxidation, which needs further study in the future.

Although we have provided evidence demonstrating that selenium supplementation can reduce side effects but does not compromise the therapeutic effect of chemoradiotherapy, the molecular mechanism remains unknown. Our trial had several limitations, including that the sample size was limited and the dosage and duration of selenium supplementation were not optimized but can be improved by longer and larger trials in the future. We will continue to follow up with our participants for a much longer time to observe the effect of selenium supplementation on the survival time and gastrointestinal side effects of our patients with cervical cancer.

## 5. Conclusion

This prospective study demonstrated that selenium supplementation could alleviate the hematologic toxicity of concurrent chemoradiotherapy, but no adverse effects were observed in the treatment of cervical cancer. Selenium supplementation does not compromise the therapeutic effect of chemoradiotherapy and could be a promising therapeutic strategy to protect against chemoradiotherapy-induced side effects.

## Data availability statement

The original contributions presented in the study are included in the article/supplementary material, further inquiries can be directed to the corresponding authors.

## Ethics statement

The studies involving human participants were reviewed and approved by the Ethics Committee of the Central Hospital of Enshi Tujia and Miao Autonomous Prefecture. The patients/participants provided their written informed consent to participate in this study.

## Author contributions

LL, CH, and ZZ: conception and design. QH, MY, XL, and PL: administrative support. BP, XF, XH, ZY, JC, DH, GS, and LW: provision of study materials or patients. QH and MY: data analysis and interpretation. MY, BP, QH, and CH: manuscript writing. All authors contributed to the article and approved the submitted version.

## Funding

This work was supported by the National Natural Science Foundation of China (No. 81660503, 82160490); Health Commission of Hubei Province Scientific Research Project (WJ2021F095); Natural Science Foundation of Enshi Tujia and Miao Autonomous Prefecture Government (E20170002, D20210033).

## Conflict of interest

The authors declare that the research was conducted in the absence of any commercial or financial relationships that could be construed as a potential conflict of interest.

## Publisher’s note

All claims expressed in this article are solely those of the authors and do not necessarily represent those of their affiliated organizations, or those of the publisher, the editors and the reviewers. Any product that may be evaluated in this article, or claim that may be made by its manufacturer, is not guaranteed or endorsed by the publisher.
